# Effectiveness of Telemedicine Interventions for Infection Prevention and Control: A Systematic Review

**DOI:** 10.7759/cureus.82075

**Published:** 2025-04-11

**Authors:** Anas E Ahmed, Omar A Alsunusi, Hussain A Alamer, Elaf A Shubayli, Haya A Alqahtani, Raghad K Juraybi, Atheer M Aboud, Mohammed S Alshihri, Aeshah H Almaghrabi, Wassal F Aljohani, Ali M Almudawi

**Affiliations:** 1 Community Medicine, Jazan University, Jazan, SAU; 2 College of Medicine, Jazan University, Jazan, SAU; 3 College of Medicine and Health Sciences, Arabian Gulf University, Manama, BHR; 4 General Practice, Primary Health Care Department, Aseer Health Cluster, Aseer, SAU; 5 College of Medicine, Taif University, Taif, SAU; 6 College of Medicine, King Khalid University, Abha, SAU

**Keywords:** digital health, healthcare-associated infections, healthcare delivery, infection control, infection prevention, infection risk reduction, patient safety, remote healthcare, telehealth interventions, telemedicine

## Abstract

Infection prevention and control is a critical aspect of healthcare delivery, especially during the ongoing challenges posed by the coronavirus pandemic. Telemedicine has emerged as a valuable strategy for reducing the risk of infection transmission while maintaining the continuity of care. This systematic review evaluates the effectiveness, benefits, and challenges of telemedicine interventions aimed at improving infection prevention and control across various healthcare settings. A comprehensive literature search was conducted in accordance with the Preferred Reporting Items for Systematic Reviews and Meta-Analyses (PRISMA) 2020 guidelines using databases including PubMed, Scopus, Web of Science, Cochrane Central Register of Controlled Trials, and the Virtual Health Library, covering studies published up to November 2024. Studies eligible for inclusion comprised randomized trials, observational studies, and mixed-methods research assessing telemedicine applications for infection control. The methodological quality of studies was assessed using established tools for qualitative and quantitative research appraisal. Five studies met the inclusion criteria and highlighted several positive outcomes. Telemedicine interventions such as mobile applications, remote video assessments of infection control practices, and virtual infection control consultations were associated with reduced infection rates, improved compliance with preventive protocols, and timely identification of procedural gaps. In inpatient settings, telemedicine also helped conserve protective equipment and reduce staff exposure. High patient satisfaction and maintained quality of care were commonly reported. However, several challenges were identified, including technical barriers, increased workload for healthcare workers, and concerns regarding data security. These findings suggest that telemedicine is a promising and adaptable solution for enhancing infection prevention efforts, but successful implementation depends on addressing operational barriers, ensuring adequate training, and strengthening infrastructure. Further rigorous research is needed to evaluate the long-term impact and cost-effectiveness of telemedicine in infection control.

## Introduction and background

The coronavirus pandemic has significantly accelerated the adoption of telemedicine, transforming healthcare delivery by enabling remote consultations and reducing the need for in-person visits. This transition has been particularly impactful in the context of infection prevention and control, where minimizing physical contact is essential for reducing transmission risks. A systematic review highlighted the role of telemedicine in preventing, diagnosing, treating, and managing diseases during the pandemic, emphasizing its effectiveness in maintaining healthcare services while adhering to social distancing measures [1]. The increased use of telemedicine services has also demonstrated its potential to advance future healthcare delivery [1].

In inpatient settings, telemedicine has been implemented as an infection control strategy to reduce healthcare workers' exposure to the coronavirus and conserve personal protective equipment. For example, a study conducted at Stanford Health Care reported that inpatient telemedicine effectively minimized exposure and reduced the use of protective equipment without significantly compromising the quality of care [2]. However, challenges such as technical limitations and the need for workflow adaptations were observed. Virtual infection prevention and control strategies have also been proposed to extend the reach of infection control specialists, particularly in low- and middle-income countries where access to specialized expertise is limited [3]. These virtual strategies are designed to provide remote support and guidance to healthcare facilities, thereby strengthening their ability to manage infectious diseases effectively.

Despite its advantages, the rapid integration of telemedicine has presented several challenges, including concerns related to data privacy, the adequacy of remote assessments, and the requirement for robust technological infrastructure [4]. A study exploring the privacy and security risks associated with telemedicine identified issues such as lack of technological familiarity, connectivity problems, and the need to safeguard patient data confidentiality [4]. Furthermore, the broader landscape of cyber risk in telemedicine encompasses system failures, inadequate informed consent procedures, complex identity and access management, increased regulatory compliance demands, and physical security threats [5].

The incorporation of telemedicine into infection control strategies has also been explored in resource-limited environments, where virtual infection prevention approaches aim to support under-resourced healthcare facilities remotely [3]. Addressing the aforementioned technological and ethical challenges is critical to fully leveraging the benefits of telemedicine in enhancing infection prevention and control.

This systematic review aims to evaluate the effectiveness and challenges of telemedicine interventions in infection prevention and control, offering insights into their impact on healthcare delivery during the coronavirus pandemic and beyond. By synthesizing existing evidence, this review seeks to inform future strategies for integrating telemedicine into infection control practices, ensuring both effectiveness and security in healthcare systems.

## Review

Literature search strategy

This review was registered with the International Prospective Register of Systematic Reviews (PROSPERO) (Reg. No. CRD42023761492). The review process adhered to the Preferred Reporting Items for Systematic Reviews and Meta-Analyses (PRISMA) guidelines [6]. A comprehensive literature search was conducted using five major electronic databases: PubMed, Virtual Health Library, Web of Science, Scopus, and the Cochrane Central Register of Controlled Trials, covering publications from the inception of each database through March 15, 2025. The search terms included combinations of “remote”, “telehealth”, and “tele” with “infection control”, using Boolean operators to optimize results. Search strategies were adapted for each database's indexing requirements. Filters were applied to include only English-language studies involving human participants. In addition, the reference lists of all included studies were manually screened to identify any relevant articles not captured by the electronic search.

Eligibility criteria

The inclusion criteria were defined using the population, intervention, comparison, outcome, and study design framework. Eligible studies were limited to English-language publications and met the following criteria: they involved individuals receiving care in healthcare settings where infection prevention and control is applicable, such as hospitals, long-term care facilities, or nursing homes; they implemented telemedicine interventions, such as remote infection control assessments, video consultations, or mobile-based monitoring tools; they included comparators, such as usual care, standard infection control protocols, or baseline (pre-intervention) data; they reported outcomes related to infection rates, adherence to infection control practices, healthcare-associated infections, or workflow impact; and they employed study designs, such as randomized controlled trials, quasi-experimental studies, cross-sectional analyses, or mixed-method evaluations. Studies published in languages other than English, conference abstracts without accessible full texts, and non-empirical publications such as editorials or narrative reviews were excluded.

Study selection

Two reviewers independently screened the titles and abstracts of all retrieved articles according to the predefined eligibility criteria. Any disagreements or discrepancies were resolved through discussion with a third reviewer until consensus was achieved. Full-text articles of all eligible studies were reviewed in detail, and data were extracted on study design, country, healthcare setting, population characteristics, type and purpose of telemedicine intervention, outcome measures, and key findings related to infection control. Discrepancies in data extraction were addressed through discussion and, if necessary, resolved with input from a third reviewer.

Quality appraisal

The methodological quality of each included study was assessed using the 2018 version of the Mixed Methods Appraisal Tool. This tool enables evaluation across diverse study designs, including qualitative, quantitative, and mixed-method research. Each study was appraised based on relevant criteria, such as the appropriateness of its research design, the adequacy of data collection methods, the clarity and relevance of analysis, and the coherence of the findings. Two reviewers conducted the appraisal independently, and any disagreements were resolved through discussion or consultation with a third reviewer. The use of this tool ensured a consistent and transparent evaluation of study quality throughout the review.

Study selection

**Figure 1:**
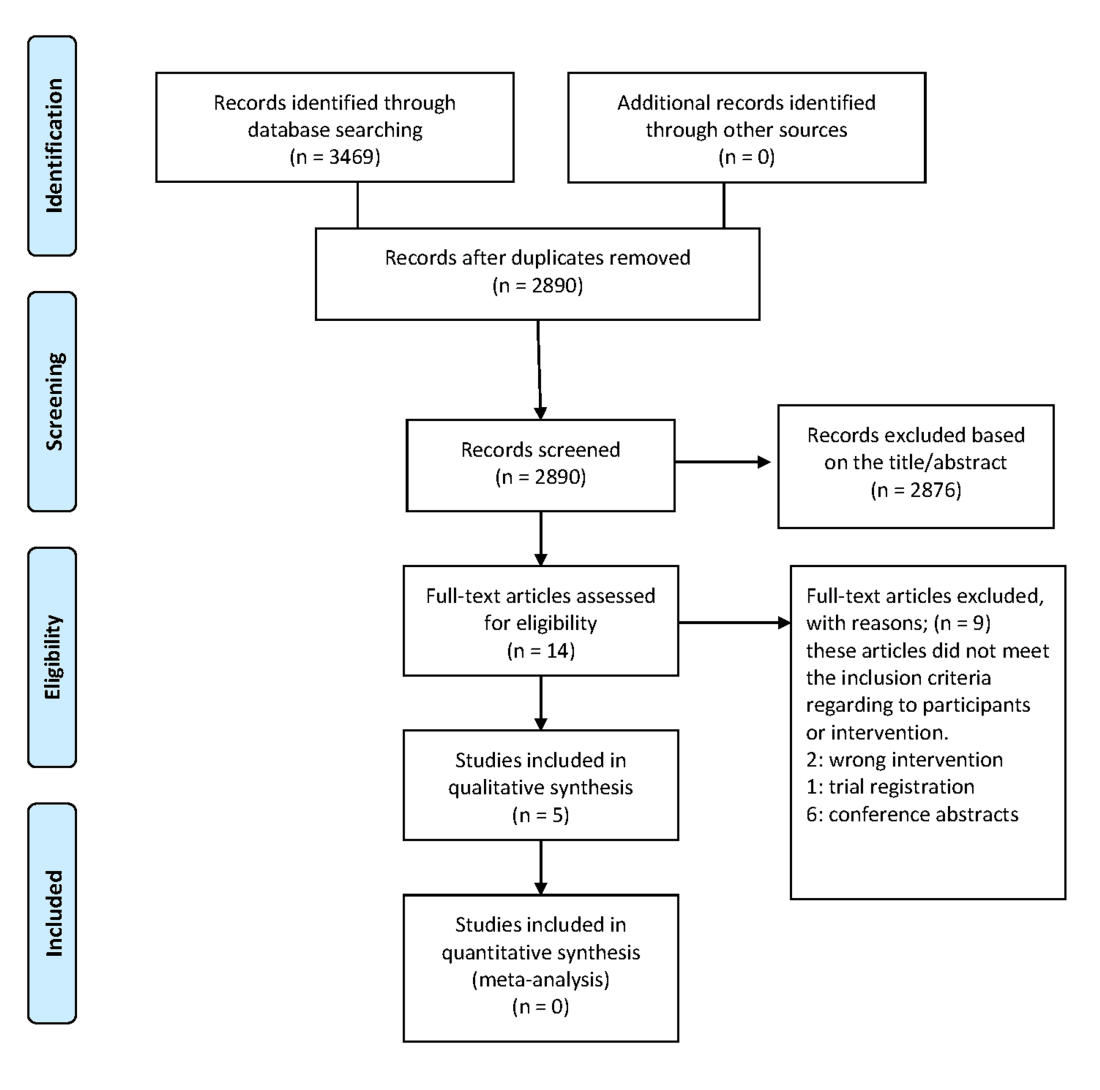
Flow diagram of the study selection process according to the Preferred Reporting Items for Systematic Reviews and Meta-Analyses (PRISMA) guidelines

The initial search across five major databases, i.e., PubMed (n = 801), Cochrane (n = 23), Virtual Health Library (n = 774), Scopus (n = 893), and Web of Science (n = 978), yielded a total of 3,469 records. No additional records were identified through other sources. After removing duplicates, 2,890 records remained for screening. During the title and abstract screening phase, 2,876 records were excluded based on irrelevance to the review topic. Fourteen full-text articles were assessed for eligibility. Of these, nine were excluded for not meeting the inclusion criteria: six were conference abstracts, two involved the wrong intervention, and one was a trial registration. Ultimately, five studies [7-11] were included in the qualitative synthesis. None met the criteria for inclusion in a quantitative synthesis. The complete selection process is illustrated in the PRISMA flow diagram (Figure 1).

Study characteristics

All five included studies were conducted in the United States and explored various telemedicine applications in healthcare settings during the COVID-19 pandemic. Despite differences in design, including pre-post quasi-experimental, cross-sectional, qualitative, and mixed-methods evaluations, they collectively examined the role of remote healthcare technologies in infection control within institutional and hospital settings (Table 1).

**Table 1:** Summary of included studies evaluating telemedicine in infection prevention and control LTCF = long-term care facility; NH = nursing home; SNF = skilled nursing facility; ALF = assisted living facility; PICC = peripherally inserted central catheter; IPC = infection prevention and control; ICAR = infection control assessment and response; TeleICAR = telephonic or video-based ICAR; ABHS = alcohol-based hand sanitizer; ABHR = alcohol-based hand rub; PPE = personal protective equipment; CLISA = central line infection score assessment. This table presents key characteristics and outcomes of studies investigating the use of telemedicine to support infection prevention and control efforts across various healthcare settings during the COVID-19 pandemic.

Author	Country	Study design	Setting	Population	Type of telemedicine	Purpose of telemedicine	Outcomes	Results
Walters et al. [7]	USA	Cross-sectional study	629 nursing homes in 19 US states	629 NHs	TeleICAR (telephone and optional video)	Assess CDC IPC guidance implementation and identify gaps	Gaps in IPC (PPE, hygiene, cohorting, education, communication); improvement post-consultation	83% had ≥1 IPC gap; 68% had core IPC gaps; 47% had cohorting/education issues; 69% made changes after TeleICAR; video revealed more real-world noncompliance
Singh RD et al. [8]	USA	Pre-post quasi-experimental cohort study	Six nursing homes in Orange County, California	719 residents, 817 PICCs	Mobile app (SAFER Lines) for photo assessments and automated physician alerts	Infection surveillance, early detection of PICC-related infection, and remote response	CLISA scores (inflammation), dressing peeling, days-to-removal, infection-related hospitalizations	↓ 73% infections (OR 0.27), ↓ 57% peeling (OR 0.43), ↓ 41% hospitalizations (OR 0.59), 95% faster line removal (from 19 to one day)
Safaeinili et al. [9]	USA	Qualitative process evaluation study	COVID-19 unit, Stanford Health Care (non-ICU)	15 clinicians: five nurses, five attendings, five residents	Tablet-based Zoom video calls (in-room devices)	Reduce COVID-19 exposure, preserve PPE, maintain care continuity for hospitalized patients	Acceptability, workflow impact, quality of care, patient-clinician communication, PPE usage	Widely adopted; reduced PPE use and exposure; care quality maintained; nurse burden noted; communication and emotional connection were key challenges
Ostrowsky et al. [10]	USA	Mixed-methods (quantitative + qualitative) pilot study	92 skilled nursing facilities (SNFs) in New York State	92 SNFs (52 reactive, 40 proactive assessments)	Remote IPC assessments (phone + video “COVIDeo”)	Assess IPC implementation in SNFs and provide real-time recommendations	IPC implementation (PPE use, ABHR, signage, cohorting, disinfection), gaps, time efficiency	100% had visitor restrictions and staff masking; 38% had ABHR at rooms; COVIDeo revealed more issues; remote = 4× more coverage than on-site visits
Singer et al. [11]	USA	Quantitative cross-sectional study	Long-term care facilities in Texas	439 tele-ICARs in 428 LTCFs (NH/SNFs, ALFs)	Remote infection control assessments (tele-ICARs via phone)	Evaluate COVID-19 IPC practices and identify gaps in LTCFs	IPC compliance (hand hygiene, PPE, dining, disinfection, cohorting, group activities)	Major IPC gaps: 21.7% lacked ABHS preference, 18.1% are unaware of disinfectant time, 17.8% didn’t stop communal dining; proactive ICARs found more gaps

Most studies were based in long-term care or skilled nursing facilities, which were especially vulnerable during the pandemic. Singh et al., Ostrowsky et al., Singer et al., and Walters et al. [7,8,10,11] focused on these environments, where telemedicine was used for infection surveillance and remote control audits. By contrast, Safaeinili et al. assessed a hospital unit designated for COVID-19 care, evaluating how video-based inpatient telemedicine influenced workflows and care delivery [9].

The type of telemedicine intervention varied across studies. Singh et al. used a mobile application (SAFER Lines) to document central line images and trigger alerts for physicians [8]. Safaeinili et al. employed a general-purpose platform, using bedside tablets and video calls to facilitate remote interactions [9]. Walters et al., Singer et al., and Ostrowsky et al. implemented remote infection control assessment models, with Ostrowsky’s study using real-time video walkthroughs for enhanced inspection [7,10,11].

Singh et al. targeted individual patient outcomes, measuring infection-related events such as catheter site inflammation, dressing integrity, and hospitalization rates [8]. Other studies focused on institutional infection prevention and control practices, assessing adherence to protocols for personal protective equipment, hand hygiene, cohorting, and staff training. Safaeinili et al. also examined human factors, including communication, clinician satisfaction, and perceived safety [9].

Outcomes varied based on study design. Singh et al. reported reductions in clinical infections and response times [8], while others emphasized improvements in compliance and quality of care. Walters et al. and Singer et al. highlighted common infection control deficiencies, noting improvements after remote consultations [7,11]. Ostrowsky et al. demonstrated that video-based assessments revealed risks not captured by checklists [10]. Safaeinili et al. documented workflow disruptions, emotional disconnects, and increased nursing workload, offering insight into the operational impact of telemedicine [9].

Quality assessment

The methodological quality of the five studies was evaluated using the Newcastle-Ottawa Scale, which assesses selection, comparability, and outcome domains. Scores range from 0 to 9, with higher scores indicating stronger methodological rigor (Table 2).

**Table 2:** Methodological quality appraisal of the included non-randomized studies This table presents the methodological quality assessment of five non-randomized studies. Each domain was scored as 1 (criterion met) or 0 (not met), except for the "Comparability of cohorts," which could receive a score of up to 2. Total scores range from 0 to 9. Power was judged qualitatively based on study design, sample size, and effect estimates.

Study	Representativeness of the exposed cohort	Selection of the non-exposed cohort	Ascertainment of exposure	Outcome not present at the start	Comparability of cohorts (0–2)	Outcome assessment	Follow-up duration	Follow-up completeness	Total (out of 9)	Power
Walters et al. [7]	1	0	1	0	2	1	0	0	7	Moderate–High
Singh et al. [8]	1	1	1	1	2	1	1	1	9	High
Safaeinili et al. [9]	1	0	1	0	1	1	0	0	5	Low–Moderate
Ostrowsky et al. [10]	1	0	1	0	1	1	0	0	6	Moderate
Singer et al. [11]	1	0	1	0	1	1	0	0	6	Moderate

Singh et al. received a score of 9 out of 9, reflecting high quality. This study demonstrated appropriate cohort selection, clearly defined exposure and outcome measures, and robust follow-up. Adjustments for confounders were made, and the study maintained high internal validity with minimal loss to follow-up [8].

Safaeinili et al., a qualitative process evaluation, scored 5 out of 9. Although the cohort and exposure were clearly defined, the study lacked a comparator group and longitudinal follow-up. Outcomes were assessed credibly, but the exploratory nature of the design limited its rating [9].

Singer et al. and Ostrowsky et al. each received 6 out of 9 stars. Both used structured assessment tools and represented their populations well but lacked defined comparators and follow-up periods. They accounted for some confounding variables but were limited by their cross-sectional nature [10,11].

Walters et al. scored 7 out of 9 and was rated as having moderate to high quality. The study used structured telemedicine assessments and included limited before-and-after comparisons. Despite lacking a formal control group, the comparative depth of analysis and attention to methodological rigor enhanced its quality rating [7].

Effect of interventions

Singh et al. evaluated the SAFER Lines mobile application across six nursing homes, focusing on infection control outcomes related to central catheter use [8]. The intervention resulted in a reduction in CLISA 2 or 3 scores from 29.9% to 12.0%, representing a 59.9% relative reduction (P < 0.001). Adjusted analyses revealed a 73% decrease in the odds of infection (odds ratio = 0.27, 95% confidence interval: 0.13-0.56, P < 0.001). Dressing complications decreased from 40.9% to 20.4%. The average time to line removal was halved and, in severe cases, dropped from 19 days to one day (P < 0.001). Hospitalizations related to infection declined from 42.9% to 31.1% (odds ratio = 0.59, 95% confidence interval: 0.42-0.83, P = 0.002). Although bacteremia rates also fell, the difference was not statistically significant. These findings underscore the potential of telemedicine to improve clinical outcomes in infection control.

Walters et al. conducted 629 remote infection control consultations across 19 states and found that 83% of facilities had at least one gap in infection prevention and control practices [7]. Common issues included inadequate use of alcohol-based hand sanitizers (39%), misunderstanding of disinfectant contact times (24%), and lack of auditing for cleaning (19%) and personal protective equipment compliance. Fourteen percent had not implemented resident masking or bundled care practices. Following consultations, 69% of facilities made improvements within one week.

Similarly, Singer et al. examined 439 long-term care facilities in Texas and found substantial deficiencies in communal dining cessation (17.8%), hand hygiene auditing (16.8%), and dedicated cohorting spaces (11.1%) [11]. Responsive assessments during outbreaks led to better adherence to federal guidelines. Assisted living facilities were significantly less likely than nursing homes to have cohorting areas (P < 0.001).

Ostrowsky et al. used both phone checklists and live video walkthroughs in 92 skilled nursing facilities [10]. Although checklist compliance appeared high, video audits uncovered issues such as the absence of hand sanitizers at room entrances (present in only 38% of facilities), and improper storage or usage of personal protective equipment. In some instances, residents were seen walking unmasked in hallways, underscoring the value of visual assessments for real-time feedback.

Safaeinili et al. explored how inpatient telemedicine altered clinical workflows in a COVID-19 unit [9]. Physicians shifted most interactions to video, reducing exposure risk and preserving supplies. Junior residents were restricted from in-room care, while nurses took on broader responsibilities, including device management, interpreter coordination, and facilitating virtual rounds.

Due to access restrictions for support staff, nurses also assumed tasks such as cleaning, food delivery, and linen changes. Remote team members relied on nurses for relaying care plans. Language barriers added delays in interpreter access, and technical issues with devices increased staff burden, diverting time from patient care.

Clinicians had mixed reactions to telemedicine. Physicians appreciated the reduced exposure risk and the efficiency of video check-ins, particularly because of their prior familiarity with such platforms [9]. However, some expressed concern over reduced bedside learning and diminished educational opportunities. Nurses reported increased workloads and minimal gains in safety or satisfaction. They were often responsible for both clinical and non-clinical tasks, including technical support.

Patients had ambivalent experiences. While telemedicine ensured continuity of care, many reported feeling isolated and disconnected due to limited in-person contact. Communication challenges, particularly in cases requiring informed consent or interpreter services, highlighted the emotional and practical limitations of remote care.

Across all studies, telemedicine was recognized as a scalable and efficient tool to support infection control, particularly during outbreaks. Its utility was evident in clinical monitoring, audits, and interdisciplinary coordination. However, consistent themes emerged around the need for dedicated training, robust technical support, and improved infrastructure. Barriers such as device maintenance, software reliability, and internet connectivity must be addressed to ensure successful implementation.

Discussion

This systematic review examined the role of telemedicine in infection prevention and control across a variety of healthcare settings, including nursing homes, long-term care facilities, and hospital units. The five included studies demonstrated that telemedicine interventions, ranging from video-based infection control assessments to mobile app surveillance tools, were effective in reducing infection-related risks, identifying gaps in infection prevention practices, and facilitating real-time responses. For example, Singh et al. [8] reported significant reductions in peripherally inserted central catheter line-associated infections and hospitalizations through the use of a photo-based mobile application. Similarly, Walters et al. [7] and Ostrowsky et al. [10] showed that remote infection control assessments uncovered widespread non-compliance with recommended protocols and prompted timely corrective actions.

A consistent pattern emerged across studies: telemedicine not only enabled remote clinical monitoring but also extended infection control expertise to resource-limited and geographically dispersed settings. Notably, studies employing video components, such as Ostrowsky et al. [10], were particularly effective in identifying issues not easily detectable through telephone-only methods, including improperly stored personal protective equipment and missing isolation signage. Likewise, Walters et al. [7] found that a significant number of facilities reported measurable improvements in personal protective equipment use and hand hygiene following remote infection control assessments. These findings are aligned with broader literature. For instance, Koonin et al. [12] demonstrated that telehealth interventions rapidly improved infection prevention practices during public health emergencies, while Drees et al. [13] emphasized the scalability and timeliness of such approaches during the COVID-19 surge.

Among the core outcomes, reduction in infection rates emerged as a primary benefit. Telemedicine interventions led to significant decreases in healthcare-associated infections by minimizing unnecessary in-person contact [14,15]. These findings are consistent with Parmar et al. [16], who documented lower infection rates in facilities using virtual infectious disease consultations, and with Koonin et al. [12], who showed that telemedicine improved outbreak containment by enabling remote patient triage and early intervention. In addition, the conservation of personal protective equipment was a key outcome in several studies. Safaeinili et al. [9] reported reduced use of protective equipment in inpatient settings due to virtual rounding, a finding echoed by guidance from the Centers for Disease Control and Prevention [14], which promoted telemedicine as a protective equipment-sparing strategy in high-transmission settings.

Another important observation was that telemedicine did not compromise the quality of care. Singh et al. [8] found no adverse effects on clinical outcomes despite reduced physical contact between patients and providers. This is supported by Burnham et al. [15], who concluded that tele-infectious disease consultations produced clinical results comparable to in-person visits. Parmar et al. [16] similarly reported that remote infectious disease management maintained clinical effectiveness while improving access to care. Furthermore, patient satisfaction remained high across interventions. The accessibility and ease of use of telemedicine were well received, as reflected in the included studies and supported by Kruse et al. [17], who found consistently high satisfaction scores due to reduced travel time and flexible scheduling.

Despite these benefits, the review also highlighted several implementation challenges. Nurses and frontline staff often experienced increased workload due to responsibilities such as device setup and coordination of remote visits [9]. Common technical barriers included unreliable Internet connectivity, limited device availability, and insufficient training on telehealth platforms. In addition, Kruse et al. [17] emphasized persistent concerns around privacy and the need for robust data security infrastructure to support widespread implementation.

## Conclusions

This systematic review demonstrates that telemedicine is a practical and impactful tool for strengthening infection prevention and control across diverse healthcare settings. The included studies showed that virtual interventions, such as remote infection control assessments, mobile surveillance tools, and teleconsultations, can effectively reduce infection rates, uncover procedural gaps, improve adherence to hygiene protocols, and support timely corrective actions. Importantly, these outcomes were achieved without compromising patient care quality, highlighting telemedicine’s ability to maintain clinical standards while minimizing in-person contact. The approach also proved valuable in optimizing the use of limited resources, including personal protective equipment, and expanding access to specialized infection control expertise in resource-constrained or geographically isolated facilities. However, the review also uncovered important operational challenges, such as staff burden, technological barriers, and privacy concerns, which must be addressed to ensure successful adoption. Overall, telemedicine represents a scalable, patient-centered, and cost-conscious solution to infection control that holds promise not only during outbreaks but also as a permanent component of routine healthcare delivery.
